# Diagnostic value of urinary protein and creatinine in combination with renal ultrasound examination in early renal damage of patients with hypertension

**DOI:** 10.12669/pjms.314.7513

**Published:** 2015

**Authors:** Jihong Zhu, Ke Wen, Hongwen He

**Affiliations:** 1 Jihong Zhu, Huaihe Hospital of Henan University, Kaifeng 475000, P. R. China; 2 Ke Wen, Huaihe Hospital of Henan University, Kaifeng 475000, P. R. China; 3 Hongwen He, Guanghua School of Stomatology, Hospital of Stomatology, Sun Yat-sen University, Guangzhou 510055, P. R. China

**Keywords:** Hypertension, Ultrasound examination, Urinary protein, Creatinine

## Abstract

**Objective:**

To evaluate the diagnostic value of urinary protein and creatinine in combination with renal Doppler ultrasound examination in early renal damage of patients with hypertension.

**Methods:**

One hundred twenty two hypertensive patients who were treated in our hospital from December 2013 to June 2014 were selected for this study, including 33, 41 and 48 cases of Stage I, Stage II and Stage III hypertension respectively. Meanwhile, 30 healthy subjects were selected as the control group. They received urinary protein, creatinine and renal Doppler ultrasound examination.

**Results:**

The urinary protein levels of Stage I, II and Stage III hypertensive patients were significantly different from that of the control group (p<0.05). Urinary creatinine levels were similar (p>0.05) in stage I and II but different from control (p<0.05) in stage III. Doppler ultrasound examination showed that Stage I hypertensive patients had similar renal longest diameter (RLD), renal parenchymal thickness (RPT) and ratio of RPT/renal sinus thickness to those of the control group (p>0.05), and RLDs of Stage II hypertensive patients and the control group were not significantly different (p>0.05).

**Conclusion:**

Urinary protein and creatinine levels in combination with renal Doppler ultrasound examination could diagnose early renal damage in patients with hypertension.

## INTRODUCTION

As a common cardiovascular disease,^1^,^2^ hypertension is mainly manifested as hemodynamic changes and injuries to many organs as it further progresses.^3^ Being one of the damaged target organs, the kidney is endangered by hypertension that increases the risk of complicated proteinuria. End-stage renal disease has been ascribed to hypertension-induced renal damage as one of the reasons,^4^-^6^ so it is important to diagnose early renal damage in hypertensive patients to improve the therapeutic effects and prognosis. Since the renal status and arterial hemodynamics of hypertensive patients change before renal function does, we herein assessed the diagnostic value of urinary protein-creatinine monitoring in combination with renal ultrasound examination in early renal damage of patients with hypertension in different stages.

## METHODS

### Baseline clinical data

This study was approved by the ethics committee of our hospital and written consent had been obtained from all patients. A total of 122 hypertensive patients who were treated in our hospital from December 2013 to June 2014 were selected for this study, including 33, 41 and 48 cases of Stage I, Stage II and Stage III hypertension respectively. The patients comprised 58 males and 64 females, aged 34-63 years old (average: 46.2±4.1). Meanwhile, 30 healthy subjects were selected as the control group, including 18 males and 12 females, aged 32-62 years old (average: 45.1±3.9). The baseline clinical data of all patients and the control group were similar.

### Methods

The levels of urinary protein and creatinine were detected. PHILIPS color Doppler ultrasound scanner with the probe frequency of 2-5 MHz was used to observe the renal status and to measure renal longest diameter (PLD), renal parenchymal thickness (RPT) and renal sinus thickness (RST). Ratio of RPT/RST was also calculated. In the meantime, blood fillings of the aorta, segmental arteries and arch arteries were observed. Blood flow parameters of the main renal artery, intrasinus segmental arteries and bilateral renal interlobar arteries were determined at about 1 cm of the renal hilum by pulse Doppler ultrasound. The angle between sound beam and direction of blood flow was <60°. Sampling was performed in triplicate at the central arterial lumen (1-3 mm), and peak systolic velocity (PSV), end-diastolic velocity (EDV) and resistive index (RI) [RI = (PSV-EDV)/PSV] of renal arteries were recorded. Renal blood-flow rate Q was calculated. Q (ml/min) = π×D2/4×V_mean_×60 [D is the inner diameter of the main renal artery, V_mean_=(PSV+EDV)/2].

### Statistical analysis

All data were input by EXCEL and processed by SPSS 18.0. The numerical data were compared by Chi-square test. The categorical data were expressed as (x±s). The data conforming to normal distribution were subjected to t test. P<0.05 was considered statistically significant.

## RESULTS

### General information

Age, gender ratio and basic diseases of hypertensive patients and the control group were similar (P>0.05).

### Urinary protein and creatinine levels

The urinary protein levels of Stage I, II and Stage III hypertensive patients were significantly different from that of the control group (p<0.05), but their urinary creatinine levels were similar (p>0.05) to control in stage I and II were significantly different from that of the control group (p<0.05) in stage III patients (Table-I).

**Table-I:** Urinary protein and creatinine levels.

Group	Case No.	Urinary protein (mg/L)	Urinary creatinine (mmol/d)
Control	30	0.59±0.49	12.11±2.42
Stage I hypertension	33	1.20±1.15∆	14.37±4.11*
Stage II hypertension	41	2.73±1.41∆	16.44±3.32*
Stage III hypertension	48	4.75±2.80∆	23.31±3.31∆

* Compared with control group, p>0.05;

∆ Compared with control group, p<0.05.

### RLD, RPT and RPT/RST

Stage I hypertensive patients had similar RLD, RPT and RPT/RST to those of the control group (p>0.05). RLDs of Stage II hypertensive patients and the control group were not significantly different (p>0.05), but with significantly different RPT and RPT/RST (p<0.05) seen in comparison with the control group. Stage III hypertensive patients had significantly different RLD, RPT and RPT/RST (p<0.05) (Table-II).

**Table-II:** Renal ultrasound results.

Group	Case No.	RLD (cm)	RPT (cm)	RPT/RST
Control	30	10.26±1.56	2.14±0.43	0.84±0.24
Stage I hypertension	33	10.25±1.46*	1.97±0.36*	0.78±0.25*
Stage II hypertension	41	9.97±1.16*	1.47±0.32∆	0.60±0.19∆
Stage III hypertension	48	9.07±0.94∆	1.20±0.21∆	0.47±0.20∆

* Compared with control group, p>0.05;

∆ Compared with control group, p<0.05.

### Detection rates of arteries by renal ultrasound

All the main renal arteries and segmental arteries were detected. As to arch arteries, the detection rate of the control group was 100%, and those of Stage I, Stage II and Stage III hypertension groups were 93.94%, 19.51% and 0% respectively (Table-III).

**Table-III:** Detection rates of renal arch arteries.

Group	Case No.	Detected	Undetected
Control	30	30 (100)	0
Stage I hypertension	33	31 (93.94)	2 (6.06)
Stage II hypertension	41	8 (19.51)	33 (80.49)
Stage III hypertension	48	0	48 (100)

### Blood flow parameters of the main renal artery and segmental arteries

PSV, EDV and RI of the main renal artery and segmental arteries in Stage I hypertension group were similar to those of the control group (p>0.05). Stage II hypertensive patients had similar PSV to that of the control group (p>0.05) but significantly different EDV and RI from those of control (p<0.05). PSV, EDV and RI of Stage III hypertension group were significantly different from those of the control group (p<0.05) (Table-IV and Table-V).

**Table-IV:** Blood flow parameters of the main renal artery.

Group	Case No.	Main renal artery		
		PSV (cm/s)	EDV (cm/s)	RI
Control	30	61.12±10.49	33.41±6.11	0.64±0.07
Stage I hypertension	33	63.08±11.32*	34.21±7.42*	0.65±0.09*
Stage II hypertension	41	58.23±8.41*	15.56±4.82∆	0.74±0.08∆
Stage III hypertension	48	44.41±9.31∆	11.14±3.16∆	0.78±0.05∆

* Compared with control group, p>0.05;

∆ Compared with control group, p<0.05.

**Table-V:** Blood flow parameters of segmental arteries.

Group	Case No.	Segmental artery		
		PSV (cm/s)	EDV (cm/s)	RI
Control	30	44.51±9.81	17.09±5.42	0.63±0.09
Stage I hypertension	33	45.18±7.42*	17.68±5.72*	0.65±0.06*
Stage II hypertension	41	40.32±8.43*	10.26±4.61∆	0.73±0.08∆
Stage III hypertension	48	33.41±7.15∆	8.64±1.86∆	0.77±0.10∆

* Compared with control group, p>0.05;

∆ Compared with control group, p<0.05.

### Renal blood-flow rates

**Fig.1:**
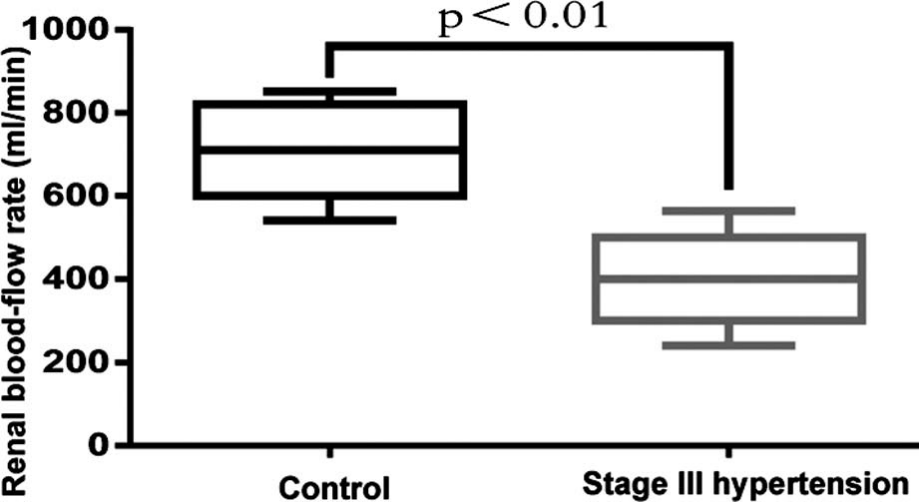
Renal blood-flow rates of control and Stage III hypertension groups.

The renal blood-flow rates of Stage I and Stage II hypertensive patients (761.48±226.58 and 669.41±149.42 ml/minute respectively) were similar to that of the control group (709.45±125.42 ml/min) (p>0.05), but the rates of Stage III hypertension (404.82±169.52 ml/min) and control groups differed significantly (p<0.01) (Fig.1).

## DISCUSSION

Hypertension is mainly clinically manifested as increase in blood pressure which, if continues, affects the structures and functions of vital organs (e.g. heart, brain and kidney) and eventually leads to their failures.^2^-^4^ The kidneys predominantly regulate the water-electrolyte balance and have several endocrine functions. Nowadays, more hypertensive patients are prone to renal damage-induced chronic renal insufficiency, 20% of whom finally suffer from end-stage renal disease.^6^-^9^ Therefore, diagnosing early renal damage of these patients plays a crucial role in improving the treatment outcomes and prognosis. In this study, 122 patients with different degrees of hypertension were subjected to urinary proteinand creatinine examinations in combination with renal ultrasound examination.

As hypertension was aggravated, the levels of urinary protein and creatinine increased. However, upon renal damage, the changes of renal status and arterial hemodynamic parameters preceded those of urinary protein and creatinine.^9^,^10^

We herein performed renal ultrasound examination to measure RLD, RPT and RST in different stages of hypertension. Stage I hypertensive patients had similar RLD, RPT and RPT/RST to those of the control group (p>0.05), and RLDs of Stage II hypertensive patients and the control group were not significantly different (p>0.05), but with significant differences between RPT and RPT/RST (p<0.05) compared with the control group. Stage III hypertensive patients had significantly different RLD, RPT and RPT/RST (p<0.05). The results suggested that the renal morphology of Stage I hypertensive patients remained almost unchanged due to mild damage to the renal parenchyma. In contrast, renal arterioles of Stage II and Stage III hypertensive patients underwent continuous sclerosis, accompanied by nephron atrophy and disappearance, obvious attenuation of the renal parenchyma, and plummet in RPT/RST.

All of the main renal arteries and segmental arteries were detected. As to arch arteries, the detection rate of the control group was 100%, and those of Stage I, Stage II and Stage III hypertension groups were 93.94%, 19.51% and 0% respectively, which were consistent with the outcomes of previous literatures.^11^,^12^

PSV, EDV and RI of the main renal artery and segmental arteries in Stage I hypertension group were similar to those of the control group (p>0.05). Stage II hypertensive patients had similar PSV to that of the control group (p>0.05) and significantly different EDV and RI from those of control (p<0.05). PSV, EDV and RI of Stage III hypertension group were significantly different from those of the control group (p<0.05). It has previously been reported that the incidence rate of renal arteriolosclerosis is positively correlated with the degree and duration of hypertension.^12^-^15^ In this study, Stage I and Stage II hypertensive patients were free from renal vascular changes and subject to moderate changes respectively, whereas Stage III ones suffered from severe renal arteriolosclerosis. Furthermore, the renal blood-flow rate of Stage II hypertension group was slightly lower than that of Stage I group (p>0.05), but the rate of Stage III group decreased significantly (p<0.05) owing to severe nephron damage.

## CONCLUSION

In summary, urinary protein and creatinine monitoring in combination with renal Doppler ultrasound examination was able to accurately, and economically diagnose early renal damage of patients with hypertension, which was of great significance for effective treatment and prognosis.
